# Association between genetic variants (rs920778, rs4759314, and rs217727) in LncRNAs and cervical cancer susceptibility in Chinese population: A systematic review and meta-analysis

**DOI:** 10.3389/fgene.2022.988207

**Published:** 2022-10-13

**Authors:** Yi Liu, Qian Zhang, Rong Ni

**Affiliations:** ^1^ Department of Gynecology, The Central Hospital of Enshi Tujia and Miao Autonomous Prefectrue, Enshi, Hubei, China; ^2^ Department of Oncology, The Central Hospital of Enshi Tujia and Miao Autonomous Prefectrue, Enshi, Hubei, China

**Keywords:** genetic variants, LncRNA, cervical cancer, SNP, susceptibility

## Abstract

**Objective:** The relationship between gene polymorphisms in long non-coding RNAs (LncRNAs) and cervical cancer susceptibility has been thoroughly analyzed; however, the conclusions are inconsistent. Therefore, this systematic review and meta-analysis aimed to accurately assess the relationship between them.

**Method:** Eligible literatures were retrieved from PubMed, Medline, China National Knowledge Infrastructure (CNKI), and WanFang databases before 1 April 2022. The odds ratios with the corresponding 95% confidence intervals were used to evaluate the strength of these relationships. Sensitivity analysis for publication bias was conducted to assess the stability and reliability of included literatures.

**Results:** A total of 59 SNPs in 11 LncRNAs were summarized for a systematic review in this study, and then, a meta-analysis of rs920778 and rs4759314 polymorphisms in HOTAIR and rs217727 polymorphisms in H19 was conducted. The results demonstrated that rs920778 and rs4759314 polymorphisms were significantly correlated with cervical cancer susceptibility. Further subgroup analysis of rs920778 polymorphism showed that both small sample size and large sample size subgroups were associated with cervical cancer susceptibility. However, no association was found between rs217727 polymorphism and cervical cancer risk in all five genetic models.

**Conclusion:** In conclusion, the rs4759314, rs920778, and rs217717 polymorphisms of HOTAIR and H19 may be associated with cervical cancer. However, the results should be interpreted with caution due to the limited sample and heterogeneity in this study. Large-scale and well-designed studies need to be practiced to validate our results.

## Introduction

Cervical cancer is the most common malignancy affecting the female reproductive system, and its incidence is increasing every year. It is the fourth most commonly diagnosed cancer worldwide and the leading cause of cancer deaths. According to statistics, there were approximately 570,000 new cases and 310,000 deaths worldwide in 2018 (Cohen et al., 2019). In the last decade, the incidence of cervical cancer has increased significantly among young people, especially among women aged 25–34 years, which has caused widespread concern (Shah et al., 2008).

Etiological studies have shown that cervical cancer is closely associated with human papillomavirus (HPV) infection, but most cases of HPV infection are self-limiting, and only a small proportion of HPV-infected individuals eventually develop cervical cancer (Foran and Brennan, 2015). Differences in host tumor susceptibility can lead to different outcomes of HPV infection (Forouzanfar et al., 2011). Therefore, the formation of cervical cancer is a multifactorial and multi-linked process, which is the result of a complex interaction between environmental factors and individual genetic factors.

Recently, long non-coding RNAs (LncRNAs) have been identified as a group of molecules closely associated with cancer heterogeneity. LncRNAs are more than 200 nucleotides in length, include polyA tails, cap structures, and promoter structures, and do not have an open reading frame (ORF) (Xing et al., 2021). Therefore, LncRNAs lack the ability to encode proteins. With the application of research technologies such as high-throughput sequencing, the biological functions of LncRNAs have been gradually discovered by scientists. lncRNAs are involved in the regulation of cell cycle; cell proliferation, apoptosis, and differentiation; epigenetic regulation; transcriptional interference; chromatin modification; and genomic imprinting (Yang et al., 2014). Furthermore, LncRNAs are closely associated with several pathological conditions such as cancer, cardiovascular disease (Liao et al., 2016), type 1 diabetes (Liu et al., 2014), rheumatoid arthritis (Yang et al., 2018), systemic lupus erythematosus (Zhao et al., 2018), and neurodegenerative diseases (Riva et al., 2016).

Single nucleotide polymorphisms (SNPs) are among the most common genetic variants in the population, with approximately 10 million SNPs in the human genome (Sachidanandam et al., 2001). SNPs in LncRNAs can affect the expression level, structure, and function of LncRNAs by interfering with the expression of the corresponding target mRNAs. In addition, SNPs can be used as biomarkers to predict cancer risk, drug resistance, clinical outcome, and prognosis (Srinivasan et al., 2016). Genome-wide association studies (GWAS) have found that SNPs in LncRNAs are significantly associated with the occurrence and progression of different types of cancers. Therefore, SNPs in LncRNAs may be related to the mechanisms of cancer susceptibility (Chen et al., 2013).

In this study, we conducted a systematic review and meta-analysis of SNPs in LncRNAs and cervical cancer susceptibility, and explored the pathogenesis of cervical cancer. SNPs in LncRNAs can be used as molecular markers to screen high-risk populations or susceptible individuals, thus providing a scientific basis for early diagnosis and treatment of cervical cancer in the future.

## Materials and methods

### Search strategy and literature selection

A systematic search of PubMed, Medline, CNKI, and Wanfang databases was conducted. Literature with a publication date before 1 April 2022 was searched. The search keywords were “cervical or CIN,” “long non-coding RNA or LncRNA,” and “polymorphisms or polymorphism”.

The following studies were included in this meta-analysis: 1) case-control studies; 2) studies in which patients in the case group were diagnosed with cervical cancer by histopathology; 3) studies that included data on first author, year of publication, ethnicity, sample size, genotype detection method, and genotype population distribution; and 4) studies published in Chinese or English.

The following studies were excluded: 1) non-cervical cancer studies; 2) studies with unclear pathological diagnosis or irregular diagnostic methods; 3) studies with incomplete genotype data; 4) studies that did not report Hardy-Weinberg equilibrium (HWE) or could not calculate HWE based on reported data; and 5) reviews, animal studies, conference papers, editorials, expert opinions, letters, bioinformatic analysis articles, and case reports.

### Quality evaluation of included literature

The Newcastle Ottawa Scale, a tool used for assessing the risk of bias in observational studies, was used to evaluate the quality of included studies, with a total score of 9 points. Studies with a score of >5 points were considered eligible for inclusion, and those who obtained a score of >7 points were considered high quality.

### Data extraction

Based on a standardized strategy, two authors (Liu and Zhang) extracted the following relevant data: first author, publishing year, country, ethnicity, LncRNAs, SNP ID, sample size, genotype frequencies, genotyping methods, *p* value for HWE in control group.

### Statistical analyses

The present meta-analysis was conducted based on the PRISMA checklists and followed its guideline (Moher et al., 2010). The odds ratios (ORs) and 95% confidence intervals (CIs) were used to describe the relationship between LncRNA SNPs and cervical cancer susceptibility. For each SNP, the ORs and 95% CIs of all five genetic models (additive model, dominant model, recessive model, homozygous model, and heterozygous model) were measured. The heterogeneity was evaluated using the Cochran’s Q test and I^2^. If I^2^ >50% and *p* < 0.1, the random effects model was used to calculate the ORs and the 95% CI; by contrast, the fixed effects model was applied. Publication bias was estimated using the *Egger*’s test. All analyses of ORs and 95% CIs were conducted using Revman (version 5.3), and sensitivity analysis was performed using the R (version 4.0) software; a two-tailed *p* value of <0.05 was considered significant.

## Result

### Characteristics of studies

**FIGURE 1:**
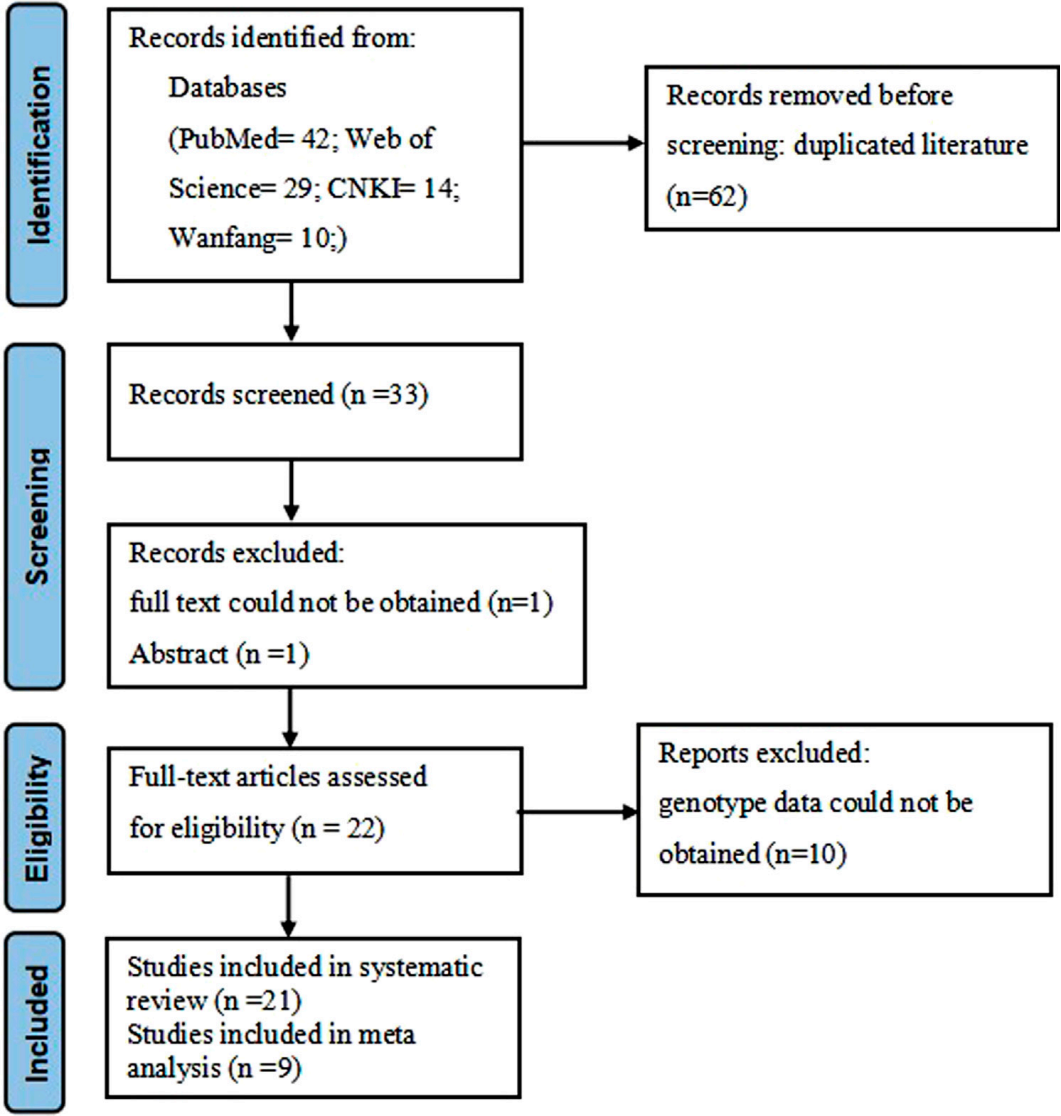
PRISMA flow chart of study selection.

A total of 95 literatures were obtained using our literature search strategy. After screening the titles and abstracts, 62 duplicate articles were removed, and the remaining 33 articles were used for subsequent analysis. Then, 12 articles were excluded, including 10 articles for which genotype data were not available, 1 article for which the abstract was available, and 1 article for which the full text was not available (Figure 1). Finally, we pooled the data of 59 SNPs from 11 different LncRNAs in 21 case-control studies (19 English and 2 Chinese publications) (Guo et al., 2015; Guo et al., 2016; Han et al., 2016; Jin et al., 2016; Qiu et al., 2016; Wu et al., 2016; He and Wei, 2017; Jin et al., 2017; Zhu et al., 2017; Wang et al., 2018; Wang and Luo, 2018; Weng et al., 2018; Huang et al., 2019; Jia et al., 2019; Gao et al., 2020; Minn et al., 2020; Wang et al., 2020; Weng et al., 2020; Sun et al., 2022; Yao et al., 2022; Yi et al., 2022). Details of these studies and the distribution of SNPs genotype are shown in Supplementary Table S1. If more than two studies reported the same LncRNA SNP, a meta-analysis was used to calculate the combined effect; therefore, a meta-analysis was conducted in three SNPs of HOTAIR (rs920778, rs4759314) and H19 (rs217727).

### HOTAIR rs920778

**FIGURE 2:**
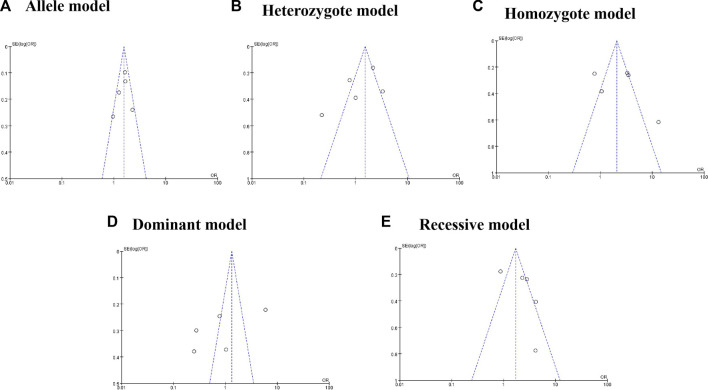
Funnel plot for rs920778 polymorphism and cancer risk.

To explore the association between HOTAIR rs920778 polymorphism and cervical cancer risk, we included five studies with 1,884 cases and 2,087 controls in our meta-analysis. The result showed that rs920778 polymorphism was correlated to higher cancer susceptibility in three genetic models (allele model, T vs. C: OR = 1.54%, 95% CI = 1.26–1.90, *p* = 0.001; homozygote model, TT *vs*. CC: OR = 1.74%, 95% CI = 1.04–2.89, *p* = 0.03; recessive model, TT *vs*. TC + CC: OR = 1.84%, 95% CI = 1.10–3.07, *p* = 0.01) (Supplementary Table S2). No publication bias was detected during the visual inspection of funnel plots (Figure 2) and was further proved by the results of *Egger*’s test (T vs. C: *P*_*Egger*_ = 0.746; TC vs. CC: *P*_*Egger*_ = 0.967; TT vs. CC: P_*Egger*_ = 0.662; TT + TC vs. CC: P_*Egger*_ = 0.841; TT vs. TC + CC: P_*Egger*_ = 0.740). Due to the presence of heterogeneity, a subgroup analysis was performed according to the sample size. An obvious association was detected in the allele, homozygote, dominant, and recessive genetic models. However, except for the allele model, the heterogeneity in the other four genetic models was still high. The result of sensitivity analysis showed no changes after deleting each study (Table 1).

**TABLE 1:** Sensitivity analysis for rs920778 polymorphism and cancer risk.

Study	OR	95%CI
Omitting Guo (China 2016)	0.61	0.43–0.85
Omitting Minn (Japan 2020)	1.64	1.42–1.89
Omitting Qiu (China 2016)	1.55	1.33–1.80
Omitting Weng (China 2016)	1.63	1.43–1.87
Omitting Yi (China 2016)	1.53	1.34–1.75

### HOTAIR rs4759314

**FIGURE 3:**
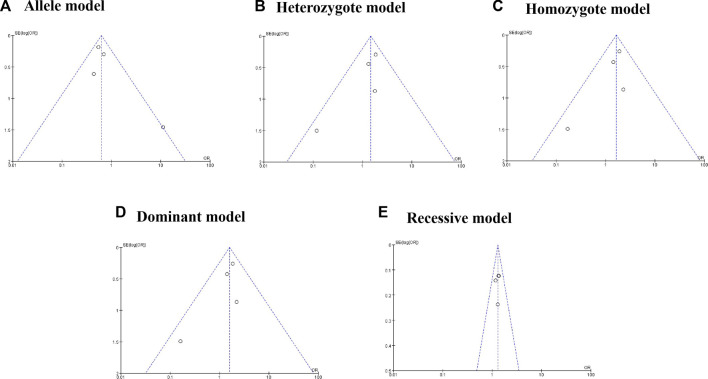
Funnel plot for rs4759314 polymorphism and cancer risk.

Four eligible studies with 2,896 patients and 3,335 controls were included in the present meta-analysis to detect the association between HOTAIR rs4759314 polymorphism and cervical cancer susceptibility. Supplementary Table S2 showed that HOTAIR rs4759314 was significantly related to cervical cancer susceptibility in three genetics models (homozygote model, GG vs. AA: OR = 1.71%, 95% CI = 1.12–2.61, *p* = 0.01; dominant model, GG + GA vs. AA: OR = 1.66%, 95% CI = 1.09–2.54, *p* = 0.02; recessive model, GG vs. GA + AA: OR = 1.31%, 95% CI = 1.14–1.51, *p* = 0.001). No heterogeneity was observed in all five genetic models. As shown in Supplementary Table S2 and Figure 3, publication bias was not found in all five genetic models after analysis using *Egger*’s test (G vs. A: P_*Egger*_ = 0.362; GA vs. AA: P_*Egger*_ = 0.084; GG vs. AA: P_*Egger*_ = 0.226; GG + GA vs. AA: P_*Egger*_ = 0.212; GG vs. GA + AA: P_*Egger*_ = 0.190) and funnel plots. Table 2 showed the results of sensitivity analysis. After eliminating Wu’s study (Wu et al., 2016), no significant heterogeneity was found.

**TABLE 2:** Sensitivity analysis for rs4759314 polymorphism and cancer risk.

Study	OR	95%CI
Omitting Guo (China 2016)	0.61	0.43–0.85
Omitting Jin (China 2019)	0.64	0.48–0.87
Omitting Weng (China 2018)	0.57	0.42–0.77
Omitting Wu (China 2016)	0.82	0.50–1.35

### H19 rs217727

Three studies with 767 cases and 809 controls were used to identify the association between H19 rs217727 and cervical cancer susceptibility in this meta-analysis. No significant association was observed between rs217727 and cervical cancer risk in all five genetic models (G vs. A: OR = 0.99%, 95% CI = 0.44–2.26, *p* = 0.99; GA vs. AA: OR = 1.00%, 95% CI = 0.60–1.67, *p* = 0.98; GG vs. AA: OR = 1.00, 95% CI = 0.37–2.69, *p* = 0.99; GG + GA vs. AA: OR = 1.00%, 95% CI = 0.45–2.21, *p* = 0.98; GG vs. GA + AA: OR = 1.00%, 95% CI = 0.72–1.38, *p* = 0.97) (Supplementary Table S2). The heterogeneity of rs217727 was high and subgroup analysis should have been performed; however, the number of included papers was very small, only 3, so subgroup analysis was not performed.

**FIGURE 4:**
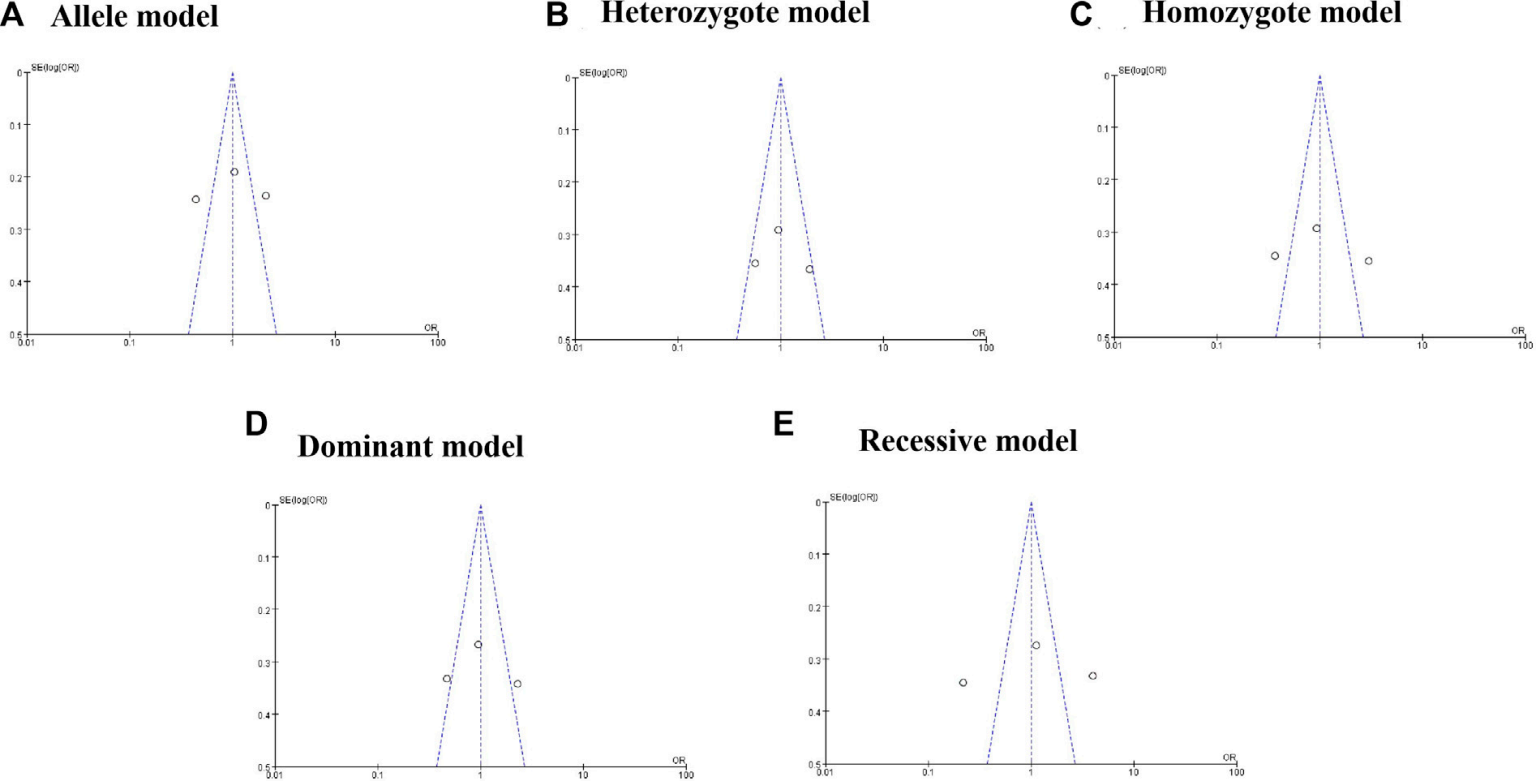
Funnel plot for rs217717 polymorphism and cancer risk.

The results of the sensitivity analysis showed no significant heterogeneity after deleting Huang’s study (Huang et al., 2019) (Table 3). Furthermore, publication bias was evaluated using the *Egger*’s test and funnel plot, and no publication bias was found (G vs. A: P_*Egger*_ = 0.339; GA vs. AA: P_*Egger*_ = 0.971; GG vs. AA: P_*Egger*_ = 0.302; GG + GA vs. AA: P_*Egger*_ = 0.117; GG vs. GA + AA: P_*Egger*_ = 0.098) (Supplementary Table S2, Figure 4).

**TABLE 3:** Sensitivity analysis for rs217717 polymorphism and cancer risk.

Study	OR	95%CI
Omitting He (China 2017)	1.38	1.04–1.85
Omitting Huang (Japan 2019)	0.98	0.71–1.34
Omitting Jin (China 2016)	0.75	0.56–0.99

## Discussion

Three SNPs in LncRNAs were evaluated from 11 published studies, namely HOTAIR rs920778, HOTAIR rs4759314, and H19 rs217727, in relation to cervical cancer susceptibility. These three SNPs were closely related to cervical cancer susceptibility. In the allele model, homozygote model, and recessive model, rs920778 showed a significant correlation with cervical cancer susceptibility; patients carrying T base may be more likely to develop cervical cancer. For rs4759314, in the homozygote model, dominant model, and recessive model, the G base may have a higher susceptibility to cervical cancer. H19 rs217727 showed no correlation with cervical cancer susceptibility in the five gene models. Previous meta-analyses have discussed SNPs and the risk of cervical cancer in different LncRNAs (Sun et al., 2022; Yao et al., 2022). This systematic review and meta-analysis was the first to summarize and evaluate all relevant literature in human studies focusing on LncRNA polymorphisms in cervical cancer. The SNPs and LncRNA cases (including patients and controls) presented in this study were more comprehensive, suggesting that the results of this study may be more accurate.

Previous studies have shown that SNPs in LncRNAs play a key role in the development of cervical cancer. LncRNA HOTAIR, encoded from the HOXC locus, is located in the region between HOXC11 and HOXC12 on chromosome 12. Previous research showed that LncRNA HOTAIR is involved in the occurrence and metastasis of cancer. The upregulation of HOTAIR expression is significantly correlated with the occurrence and metastasis of liver cancer, ovarian cancer, pancreatic cancer, neuroblastoma, and other cancer types (Qu et al., 2019). Multiple SNPs in HOTAIR affect the tumor susceptibility by changing its secondary structure. Qiu et al. reported that homozygous TT genotype of rs920778 polymorphism significantly increased the risk of cervical cancer. Allele T carriers were associated with an increased risk of cervical cancer compared with allele C (Qiu et al., 2016). Guo et al. found that homozygous TT genotype with rs920778 polymorphism is significantly correlated with the tumor-node-metastasis stage in cervical cancer patients. However, other studies reported conflicting results (Guo et al., 2016). Weng et al. (2018) demonstrated that the overall survival of cervical cancer patients with CC genotype carrying rs920778 polymorphism was poor, and no genotypic differences were noted between cervical cancer patients, invasive cancer patients, and normal controls. An intron enhancer was found between +1719bp and + 2353bp at the transcription start site of HOTAIR gene, and an rs920778 polymorphism was located in this region. rs920778 polymorphism can regulate the expression of HOTAIR RNA in esophagus cancer cell lines and normal esophageal tissue specimens by changing the enhancer activity in cells, and the expression of HOTAIR is higher in T allele carriers (Zhang et al., 2014). This meta-analysis found that rs920778 was significantly associated with a higher susceptibility to cervical cancer, and three genotypes found that carriers of the T allele had a higher risk of developing cervical cancer. We speculated that the increased expression of HOTAIR in cervical cells of patients carrying the T allele activates multiple pro-cancer signaling pathways or carcinogenic molecules, thus inducing the occurrence of cervical cancer.

In addition, the G allele of rs4759314 polymorphism can also promote the transcriptional activity of HOTAIR promoter. The G allele transfected with rs4759314 polymorphism could significantly improve the luciferase activity of cells, suggesting that the G allele of rs4759314 polymorphism could improve the RNA level of HOTAIR (Deng et al., 2020). Wu et al. found that the G allele of rs4759314 polymorphism increases the risk of ovarian cancer (Wu et al., 2016). However, Weng et al. (2018) reported that the three genotypes of rs4759314 polymorphism did not differ between cervical cancer patients, invasive cancer patients, and normal controls. The results of our existing meta-analysis suggest that individuals carrying the G allele of rs4759314 polymorphism may have a higher susceptibility to cervical cancer. We hypothesized that the G allele of rs4759314 polymorphism promoted the transcriptional activity of HOTAIR promoter in cervical cells and then increased the RNA level of HOTAIR, leading to the occurrence of cancer.

The long non-coding RNA gene H19 (LncRNA gene H19) is located in the telomere region of chromosome 11p15.5. H19 is overexpressed in a variety of cancer types (Ghafouri-Fard et al., 2020). Furthermore, H19 expression is associated with histological grade, clinical stage, and lymph node metastasis status and can be used as a biomarker to predict the pathological features. rs217727 is located in the fifth exon of H19 gene (Lin et al., 2017; Abdollahzadeh and Ghorbian, 2019). The CT or TT genotype of rs217727 polymorphism was helpful in improving the expression level of H19 (Xia et al., 2016). rs217727 increased the risk of breast, stomach, and cervical cancer (Yang et al., 2015; Jin et al., 2016). However, another study showed that rs217727 was not associated with colorectal cancer risk (Li et al., 2016). In this meta-analysis, the various genotypes of rs217727 polymorphism were not significantly associated with the risk of developing cervical cancer.

Our meta-analysis has several advantages. First, this meta-analysis was the first to examine the association between LncRNA polymorphisms and cervical cancer susceptibility. Second, according to the methodological quality assessment, the included studies were of high quality. Furthermore, no restriction was set in the literature retrieval strategy; hence, the selection bias was well-controlled.

Our meta-analysis has several limitations. First, the sample size of this study was limited, which may reduce the accuracy of the analysis. Therefore, these data need to be validated in studies with a larger sample size. Second, the search was only performed in English and Chinese databases, which may have affected the search results. If literatures in other languages are included, this study may have been more comprehensive. Finally, heterogeneity was still observed after the subgroup analysis; therefore, our conclusions should be treated with caution.

In summary, rs920778 and rs4759314 polymorphisms are significantly associated with increased risk of cervical cancer, while rs217727 polymorphisms may not be associated with cervical cancer susceptibility. However, due to the small number of studies included, there is not enough data to fully confirm the association between cervical cancer and these polymorphisms; hence, the results should be interpreted with caution.

## Data Availability

The original contributions presented in the study are included in the article/Supplementary Material, further inquiries can be directed to the corresponding author.
