# Supplementary material for: Association between genetic variants (rs920778, rs4759314, and rs217727) in LncRNAs and cervical cancer susceptibility in Chinese population: A systematic review and meta-analysis
Source: Front Genet. 2022 Oct 13;13:988207. doi: 10.3389/fgene.2022.988207 (PMC9608570; doi:10.3389/fgene.2022.988207)
Supplement: Supplementary file 1 [file Table1.docx]

**Table 1 Description of the SNPs articles included in this meta-analysis**

| First  author | Year | Country | Ethnicity | LncRNAs | SNP ID | Case | | | Control | | | Sample size  (case/control) | Genotyping | P for  HWE | OR | P value | Quality | Reference |
| --- | --- | --- | --- | --- | --- | --- | --- | --- | --- | --- | --- | --- | --- | --- | --- | --- | --- | --- |
| Yao | 2022 | China | Chinese | ANRIL | rs1333048 | CC | AC | AA | CC | AC | AA |  | TaqMan assay |  |  |  | 7 | 16 |
|  |  |  |  |  |  | 160 | 298 | 129 | 149 | 351 | 161 | 587/661 |  | >0.05 | 0.74 (0.54–1.02) | 0.130 |  |  |
|  |  |  |  |  | rs4977574 | AA | AG | GG | AA | AG | GG |  |  |  |  |  |  |  |
|  |  |  |  |  |  | 131 | 304 | 152 | 184 | 354 | 132 | 587/670 |  | >0.05 | 1.63 (1.18–2.26) | 0.024 |  |  |
|  |  |  |  |  | rs1333045 | CC | CT | TT | CC | CT | TT |  |  |  |  |  |  |  |
|  |  |  |  |  |  | 163 | 302 | 122 | 152 | 353 | 156 | 587/661 |  | >0.05 | 0.72 (0.52–1.00) | 0.045 |  |  |
|  |  |  |  |  | rs10757278 | AA | AG | GG | AA | AG | GG |  |  |  |  |  |  |  |
|  |  |  |  |  |  | 137 | 293 | 157 | 164 | 353 | 144 | 587/661 |  | >0.05 | 1.32 (0.95–1.81) | 0.550 |  |  |
|  |  |  |  |  |  |  |  |  |  |  |  |  |  |  |  |  |  |  |
| Gao | 2020 | China | Chinese | CASC15 | rs1555529 | GG | GA | AA | GG | GA | AA |  | MassARRAY |  |  |  | 7 | 17 |
|  |  |  |  |  |  | 212 | 213 | 69 | 198 | 234 | 72 | 494/504 |  | >0.05 | 0.92 (0.77–1.10) | 0.360 |  |  |
|  |  |  |  |  | rs7740084 | AA | AG | GG | AA | AG | GG |  |  |  |  |  |  |  |
|  |  |  |  |  |  | 158 | 233 | 103 | 153 | 250 | 99 | 494/502 |  | >0.05 | 0.99 (0.83–1.18) | 0.933 |  |  |
|  |  |  |  |  | rs1928168 | TT | TC | CC | TT | TC | CC |  |  |  |  |  |  |  |
|  |  |  |  |  |  | 321 | 152 | 21 | 322 | 166 | 15 | 494/503 |  | >0.05 | 1.01 (0.81–1.26) | 0.932 |  |  |
|  |  |  |  |  | rs12212674 | TT | TA | AA | TT | TA | AA |  |  |  |  |  |  |  |
|  |  |  |  |  |  | 353 | 129 | 10 | 387 | 113 | 4 | 522/504 |  | >0.05 | 1.31 (1.01–1.69) | 0.041 |  |  |
|  |  |  |  |  | rs4712653 | CC | CT | TT | CC | CT | TT |  |  |  |  |  |  |  |
|  |  |  |  |  |  | 260 | 198 | 36 | 270 | 201 | 31 | 494/502 |  | >0.05 | 1.06 (0.87–1.29) | 0.568 |  |  |
|  |  |  |  |  | rs9393266 | CC | CT | TT | CC | CT | TT |  |  |  |  |  |  |  |
|  |  |  |  |  |  | 122 | 260 | 106 | 123 | 260 | 118 | 488/501 |  | >0.05 | 0.96 (0.80–1.14) | 0.612 |  |  |
|  |  |  |  |  |  |  |  |  |  |  |  |  |  |  |  |  |  |  |
| Zhu | 2017 | China | Chinese | GAS5 | rs145204276 | ins/ins | ins/del | del/del | ins/ins | ins/del | del/del |  | TaqMan assay |  |  |  | 8 | 18 |
|  |  |  |  |  |  | 364 | 433 | 123 | 486 | 459 | 73 | 920/1018 |  | >0.05 | 1.27 (1.04–1.52) | 0.020 |  |  |
| Weng | 2020 | China | Chinese | GAS5 | rs145204276 | ins/ins | ins/del | del/del | ins/ins | ins/del | del/del |  | TaqMan assay |  |  |  | 7 | 19 |
|  |  |  |  |  |  | 83 | 102 | 23 | 119 | 138 | 50 | 208/307 |  | >0.05 | 0.66 (0.37-1.16) | 0.151 |  |  |
|  |  |  |  |  | rs55829688 | TT | TC | CC | TT | TC | CC |  | TaqMan assay |  |  |  |  |  |
|  |  |  |  |  |  | 99 | 86 | 13 | 148 | 121 | 26 | 198/295 |  | >0.05 | 0.75 (0.37-1.53) | 0.423 |  |  |
|  |  |  |  |  |  |  |  |  |  |  |  |  |  |  |  |  |  |  |
| Jin | 2016 | China | Chinese | H19 | rs217727 | GG | GA | AA | GG | GA | AA |  | MassARRAY |  |  |  | 7 | 20 |
|  |  |  |  |  |  | 169 | 99 | 16 | 117 | 103 | 26 | 284/246 |  | >0.05 | 1.53 (1.17–2.02) | 0.002 |  |  |
| He | 2017 | China | Chinese | H19 | rs217727 | GG | GA | AA | GG | GA | AA |  | PCR-RFLP |  |  |  | 7 | 21 |
|  |  |  |  |  |  | 118 | 104 | 28 | 149 | 87 | 14 | 250/250 |  | >0.05 | 1.65 (1.14-2.28) | 0.006 |  |  |
| Huang | 2019 | China | Chinese | H19 | rs3024270 | GG | GC | CC | GG | GC | CC |  | TaqMan assay |  |  |  | 6 | 22 |
|  |  |  |  |  |  | 60 | 120 | 51 | 95 | 150 | 71 | 231/316 |  | >0.05 | 0.88 (0.54-1.43) | 0.512 |  |  |
|  |  |  |  |  | rs2839698 | CC | CT | TT | CC | CT | TT |  |  |  |  |  |  |  |
|  |  |  |  |  |  | 115 | 99 | 20 | 154 | 134 | 30 | 234/318 |  | >0.05 | 089 (0.48-1.65) | 0.936 |  |  |
|  |  |  |  |  | rs3741219 | AA | AG | GG | AA | AG | GG |  |  |  |  |  |  |  |
|  |  |  |  |  |  | 112 | 100 | 21 | 152 | 130 | 32 | 233/314 |  | >0.05 | 0.89 (0.49-1.63) | 0.875 |  |  |
|  |  |  |  |  | rs2107425 | CC | CT | TT | CC | CT | TT |  |  |  |  |  |  |  |
|  |  |  |  |  |  | 88 | 107 | 38 | 109 | 155 | 48 | 233/312 |  | >0.05 | 0.98 (0.59-1.63) | 0.684 |  |  |
|  |  |  |  |  | rs217727 | GG | GA | AA | GG | GA | AA |  |  |  |  |  |  |  |
|  |  |  |  |  |  | 102 | 103 | 28 | 135 | 139 | 39 | 233/313 |  | >0.05 | 0.95 (0.55-1.65) | 0.875 |  |  |
|  |  |  |  |  |  |  |  |  |  |  |  |  |  |  |  |  |  |  |
| Guo | 2016 | China | Chinese | HOTAIR | rs920778 | TT | TC | CC | TT | TC | CC |  | MALDI-TOF Mass Spectrometry |  |  |  | 8 | 23 |
|  |  |  |  |  |  | 52 | 189 | 269 | 30 | 235 | 448 | 510/713 |  | >0.05 | 2.88 (1.76–4.71) | 0.001 |  |  |
|  |  |  |  |  | rs1899663 | GG | GT | TT | GG | GT | TT |  |  |  |  |  |  |  |
|  |  |  |  |  |  | 356 | 146 | 8 | 509 | 191 | 13 | 510/713 |  | >0.05 | 0.88 (0.35–2.29) | 0.645 |  |  |
|  |  |  |  |  | rs4759314 | AA | AG | GG | AA | AG | GG |  |  |  |  |  |  |  |
|  |  |  |  |  |  | 378 | 121 | 11 | 544 | 158 | 11 | 510/713 |  | >0.05 | 1.41 (0.59–3.58) | 0.316 |  |  |
| Qiu | 2016 | China | Chinese | HOTAIR | rs920778 | TT | TC | CC | TT | TC | CC |  | TaqMan assay |  |  |  | 7 | 24 |
|  |  |  |  |  |  | 47 | 78 | 90 | 54 | 150 | 226 | 215/430 |  | >0.05 | 2.186 (1.378–3.466) | 0.003 |  |  |
| Wu | 2016 | China | Chinese | HOTAIR | rs4759314 | AA | AG | GG | AA | AG | GG |  |  |  |  |  |  | 25 |
|  |  |  |  |  |  | 819 | 140 | 41 | 852 | 125 | 23 | 1000/1000 |  | >0.05 | 1.85 (1.11–3.09) | 0.007 | 8 |  |
|  |  |  |  |  | rs7958904 | GG | GC | CC | GG | GC | CC |  |  |  |  |  |  |  |
|  |  |  |  |  |  | 594 | 355 | 51 | 533 | 380 | 87 | 1000/1000 |  | >0.05 | 0.53 (0.37–0.75) | 0.000 |  |  |
|  |  |  |  |  | rs874945 | GG | GA | AA | GG | GA | AA |  |  |  |  |  |  |  |
|  |  |  |  |  |  | 665 | 283 | 52 | 677 | 279 | 44 | 1000/1000 |  | >0.05 | 1.20 (0.79–1.82) | 0.382 |  |  |
| Jin | 2017 | China | Chinese | HOTAIR | rs4759314 | AA | AG | GG | AA | AG | GG |  | TaqMan assay |  |  |  | 8 | 26 |
|  |  |  |  |  |  | 1012 | 158 | 4 | 1162 | 140 | 2 | 1174/1304 |  | >0.05 | 1.50 (0.26–8.69) | 0.071 |  |  |
|  |  |  |  |  | rs7958904 | GG | GC | CC | GG | GC | CC |  |  |  |  |  |  |  |
|  |  |  |  |  |  | 640 | 427 | 86 | 735 | 494 | 63 | 1153/1292 |  | >0.05 | 1.58 (1.10–2.28) | 0.029 |  |  |
|  |  |  |  |  | rs874945 | GG | GA | AA | GG | GA | AA |  |  |  |  |  |  |  |
|  |  |  |  |  |  | 745 | 383 | 43 | 852 | 394 | 43 | 1171/1289 |  | >0.05 | 1.26 (0.79–2.02) | 0.434 |  |  |
| Weng | 2018 | China | Chinese | HOTAIR | rs920778 | TT | TC | CC | TT | TC | CC |  | TaqMan assay |  |  |  | 7 | 27 |
|  |  |  |  |  |  | 107 | 92 | 13 | 165 | 134 | 19 | 212/318 |  | >0.05 | 1.06 (0.50-2.23) | 0.950 |  |  |
|  |  |  |  |  | rs12427129 | CC | CT | TT | CC | CT | TT |  |  |  |  |  |  |  |
|  |  |  |  |  |  | 181 | 29 | 2 | 275 | 42 | 1 | 212/318 |  | >0.05 | 3.04 (0.27-33.76) | 0.629 |  |  |
|  |  |  |  |  | rs1899663 | GG | GT | TT | GG | GT | TT |  |  |  |  |  |  |  |
|  |  |  |  |  |  | 140 | 67 | 5 | 209 | 101 | 8 | 212/318 |  | >0.05 | 0.93 (0.30-2.91) | 0.992 |  |  |
|  |  |  |  |  | rs4759314 | AA | AG | GG | AA | AG | GG |  |  |  |  |  |  |  |
|  |  |  |  |  |  | 171 | 41 | 0 | 266 | 48 | 4 | 212/318 |  | >0.05 | 1.33 (0.84-2.10) | 0.124 |  |  |
| Jia | 2019 | China | Chinese | HOTAIR | rs10783618 | CC | CT | TT | CC | CT | TT |  | Mass ARRAY |  |  |  | 7 | 28 |
|  |  |  |  |  |  | 757 | 510 | 84 | 822 | 559 | 107 | 1351/1488 |  | >0.05 | 0.85 (0.63–1.15) | 0.300 |  |  |
|  |  |  |  |  | rs11170776 | GG | GT | TT | GG | GT | TT |  |  |  |  |  |  |  |
|  |  |  |  |  |  | 848 | 391 | 52 | 959 | 466 | 69 | 1291/1494 |  | >0.05 | 0.85 (0.59–1.24) | 0.400 |  |  |
|  |  |  |  |  | rs117363442 | CC | CG | GG | CC | CG | GG |  |  |  |  |  |  |  |
|  |  |  |  |  |  | 1 130 | 214 | 11 | 1 252 | 236 | 8 | 1355/1496 |  | >0.05 | 1.52 (0.61–3.80) | 0.370 |  |  |
|  |  |  |  |  | rs35643724 | GG | GA | AA | GG | GA | AA |  |  |  |  |  |  |  |
|  |  |  |  |  |  | 750 | 518 | 86 | 877 | 541 | 77 | 1354/1495 |  | >0.05 | 1.31 (0.95–1.80) | 0.110 |  |  |
|  |  |  |  |  | rs4564384 | CC | CT | TT | CC | CT | TT |  |  |  |  |  |  |  |
|  |  |  |  |  |  | 1 139 | 193 | 15 | 1 224 | 260 | 11 | 1347/1495 |  | >0.05 | 1.47 (0.67–3.20) | 0.340 |  |  |
|  |  |  |  |  | rs894739 | CC | CT | TT | CC | CT | TT |  |  |  |  |  |  |  |
|  |  |  |  |  |  | 859 | 420 | 65 | 995 | 437 | 62 | 1344/1494 |  | >0.05 | 1.21 (0.85–1.74) | 0.290 |  |  |
|  |  |  |  |  | rs920777 | GG | GA | AA | GG | GA | AA |  |  |  |  |  |  |  |
|  |  |  |  |  |  | 1 232 | 119 | 5 | 1 328 | 166 | 2 | 1356/1496 |  | >0.05 | 2.70 (0.52–13.92) | 0.290 |  |  |
| Minn | 2020 | Japan | Japanese | HOTAIR | rs920778 | TT | TC | CC | TT | TC | CC |  | TaqMan assay |  |  |  | 7 | 29 |
|  |  |  |  |  |  | 470 | 318 | 39 | 306 | 208 | 32 | 827/546 |  | >0.05 | 0.79 (0.49–1.29) | 0.349 |  |  |
| Yi | 2022 | China | Chinese | HOTAIR | rs920778 | TT | TC | CC | TT | TC | CC |  | TaqMan assay |  |  |  | 7 | 30 |
|  |  |  |  |  |  | 35 | 55 | 30 | 10 | 38 | 32 | 120/80 |  | >0.05 | 8.153 | 0.004 |  |  |
|  |  |  |  |  |  |  |  |  |  |  |  |  |  |  |  |  |  |  |
| Wang | 2020 | China | Chinese | HULC | rs3005167 | GG | GC | CC | GG | GC | CC |  | TaqMan assay |  |  |  | 7 | 31 |
|  |  |  |  |  |  | 161 | 135 | 26 | 171 | 127 | 33 | 322/331 |  | >0.05 | 0.85 (0.49–1.49) | 0.570 |  |  |
|  |  |  |  |  | rs7770772 | GG | GC | CC | GG | GC | CC |  |  |  |  |  |  |  |
|  |  |  |  |  |  | 190 | 116 | 16 | 203 | 108 | 20 | 322/331 |  | >0.05 | 0.87 (0.44–1.74) | 0.7000 |  |  |
|  |  |  |  |  | rs1041279 | CC | CG | GG | CC | CG | GG |  |  |  |  |  |  |  |
|  |  |  |  |  |  | 93 | 143 | 86 | 121 | 147 | 63 | 322/331 |  | >0.05 | 1.79 (1.17–2.73) | 0.007 |  |  |
|  |  |  |  |  |  |  |  |  |  |  |  |  |  |  |  |  |  |  |
| Wang | 2018 | China | Chinese | LINC00673 | rs11655237 | GG | GA | AA | GG | GA | AA |  | TaqMan assay |  | 1.58 (1.05-2.37) | 0.028 | 7 | 32 |
|  |  |  |  |  |  | 561 | 374 | 65 | 615 | 338 | 47 | 1000/1000 |  | >0.05 |  |  |  |  |
|  |  |  |  |  |  |  |  |  |  |  |  |  |  |  |  |  |  |  |
| Wang | 2018 | China | Chinese | MALAT1 | rs546202 | AA | AG | GG | AA | AG | GG |  | MassARRAY |  |  |  | 7 | 33 |
|  |  |  |  |  |  | 59 | 79 | 26 | 155 | 205 | 68 | 164/428 |  | >0.05 | 1.00 (0.58–1.73) | 0.981 |  |  |
|  |  |  |  |  | rs593982 | AA | AG | GG | AA | AG | GG |  |  |  |  |  |  |  |
|  |  |  |  |  |  | 22 | 79 | 63 | 55 | 190 | 183 | 164/428 |  | >0.05 | 0.86 (0.49–1.52) | 0.607 |  |  |
| Jia | 2019 | China | Chinese | MALAT1 | rs4102217 | GG | GC | CC | GG | GC | CC |  | Mass ARRAY |  |  |  | 8 | 28 |
|  |  |  |  |  |  | 964 | 347 | 39 | 1 108 | 360 | 28 | 1350/1496 |  | >0.05 | 1.60 (0.98–2.62) | 0.06 |  |  |
|  |  |  |  |  | rs648578 | CC | CT | TT | CC | CT | TT |  |  |  |  |  |  |  |
|  |  |  |  |  |  | 1 134 | 208 | 11 | 1 258 | 224 | 14 | 1353/1496 |  | >0.05 | 1.11 (0.53–2.34) | 0.790 |  |  |
|  |  |  |  |  | rs1787666 | AA | AC | CC | AA | AC | CC |  |  |  |  |  |  |  |
|  |  |  |  |  |  | 778 | 489 | 78 | 811 | 561 | 116 | 1345/1488 |  | >0.05 | 0.70 (0.52–0.95) | 0.02 |  |  |
| Sun | 2022 | China | Chinese | MALAT1 | rs3200401 | CC | CT | TT | CC | CT | TT |  | TaqMan assay |  | 1.10 (0.36-3.35) | 0.865 | 8 | 34 |
|  |  |  |  |  |  | 137 | 62 | 6 | 176 | 85 | 7 | 205/268 |  | >0.05 |  |  |  |  |
|  |  |  |  |  | rs619586 | AA | AG | GG | AA | AG | GG |  |  |  |  |  |  |  |
|  |  |  |  |  |  | 182 | 23 | 0 | 230 | 38 | 0 | 205/268 |  | >0.05 | 0.77 (0.44-1.33) | 0.342 |  |  |
|  |  |  |  |  | rs1194338 | CC | CA | AA | CC | CA | AA |  |  |  |  |  |  |  |
|  |  |  |  |  |  | 85 | 92 | 28 | 112 | 127 | 29 | 205/268 |  | >0.05 | 1.27 (0.71-2.30) | 0.425 |  |  |
| Yao | 2022 | China | Chinese | MALAT1 | rs11227209 | CC | CG | GG | CC | CG | GG |  | TaqMan assay |  |  |  | 7 | 16 |
|  |  |  |  |  |  | 528 | 59 | 0 | 598 | 61 | 2 | 587/661 |  | >0.05 | 1.10 (0.76–1.61) | 0.260 |  |  |
|  |  |  |  |  | rs619586 | AA | AG | GG | AA | AG | GG |  |  |  |  |  |  |  |
|  |  |  |  |  |  | 490 | 89 | 8 | 555 | 100 | 6 | 587/661 |  | >0.05 | 1.52 (0.52–4.43) | 0.740 |  |  |
|  |  |  |  |  | rs664589 | CC | CG | GG | CC | CG | GG |  |  |  |  |  |  |  |
|  |  |  |  |  |  | 501 | 84 | 2 | 564 | 90 | 7 | 587/661 |  | >0.05 | 0.32 (0.07–1.55) | 0.290 |  |  |
|  |  |  |  |  | rs3200401 | CC | CT | TT | CC | CT | TT |  |  |  |  |  |  |  |
|  |  |  |  |  |  | 384 | 178 | 25 | 484 | 163 | 14 | 587/661 |  | >0.05 | 2.26 (1.16–4.42) | 0.004 |  |  |
|  |  |  |  |  |  |  |  |  |  |  |  |  |  |  |  |  |  |  |
| Han | 2016 | China | Chinese | PAX-AS1 | rs4848320 | CC | CT | TT | CC | CT | TT |  | MassARRAY |  |  |  | 7 | 35 |
|  |  |  |  |  |  | 1034 | 368 | 29 | 1069 | 407 | 50 | 1431/1526 |  | >0.05 | 0.60 (0.36–0.93) | 0.031 |  |  |
|  |  |  |  |  | rs1110839 | TT | TG | GG | TT | TG | GG |  |  |  |  |  |  |  |
|  |  |  |  |  |  | 686 | 627 | 132 | 670 | 674 | 170 | 1445/1514 |  | >0.05 | 0.76 (0.59–0.97) | 0.031 |  |  |
|  |  |  |  |  |  |  |  |  |  |  |  |  |  |  |  |  |  |  |
| Wang | 2018 | China | Chinese | THRIL | rs11057978 | AA | AG | GG | AA | AG | GG |  | MassARRAY |  |  |  | 7 | 33 |
|  |  |  |  |  |  | 144 | 20 | 0 | 389 | 39 | 0 | 164/428 |  | >0.05 | 1.39 (0.78–2.45) | 0.264 |  |  |
|  |  |  |  |  | rs10846806 | CC | CT | TT | CC | CT | TT |  |  |  |  |  |  |  |
|  |  |  |  |  |  | 4 | 42 | 118 | 13 | 98 | 317 | 164/428 |  | >0.05 | 0.86 (0.49–1.52) | 0.607 |  |  |
|  |  |  |  |  | rs7133268 | AA | AG | GG | AA | AG | GG |  |  |  |  |  |  |  |
|  |  |  |  |  |  | 68 | 65 | 1 | 145 | 215 | 68 | 134/428 |  | >0.05 | 1.21 (0.39–3.78) | 0.743 |  |  |
|  |  |  |  |  | rs731388 | CC | CT | TT | CC | CT | TT |  |  |  |  |  |  |  |
|  |  |  |  |  |  | 69 | 75 | 20 | 173 | 208 | 47 | 164/428 |  | >0.05 | 0.97 (0.58–1.64) | 0.031 |  |  |
|  |  |  |  |  | rs7132145 | AA | AG | GG | AA | AG | GG |  |  |  |  |  |  |  |
|  |  |  |  |  |  | 3 | 52 | 109 | 14 | 116 | 298 | 164/428 |  | >0.05 | 1.07 (0.59–1.93) | 0.830 |  |  |
|  |  |  |  |  | rs10846807 | CC | CT | TT | CC | CT | TT |  |  |  |  |  |  |  |
|  |  |  |  |  |  | 95 | 62 | 7 | 238 | 167 | 23 | 164/428 |  | >0.05 | 0.76 (0.32–1.84) | 0.545 |  |  |
|  |  |  |  |  | rs11613364 | CC | CT | TT | CC | CT | TT |  |  |  |  |  |  |  |
|  |  |  |  |  |  | 130 | 33 | 1 | 336 | 89 | 3 | 164/428 |  | >0.05 | 0.86 (0.09–8.36) | 0.898 |  |  |
|  |  |  |  |  | rs7975925 | AT | AT | TT | AT | AT | TT |  |  |  |  |  |  |  |
|  |  |  |  |  |  | 130 | 30 | 4 | 344 | 78 | 6 | 164/428 |  | >0.05 | 1.76 (0.49–6.35) | 0.385 |  |  |
|  |  |  |  |  | rs11615831 | AT | AT | TT | AT | AT | TT |  |  |  |  |  |  |  |
|  |  |  |  |  |  | 6 | 61 | 97 | 20 | 154 | 154 | 164/428 |  | >0.05 | 1.27 (0.50–3.27) | 0.615 |  |  |
|  |  |  |  |  | rs6488972 | GG | GT | TT | GG | GT | TT |  |  |  |  |  |  |  |
|  |  |  |  |  |  | 65 | 73 | 26 | 164 | 200 | 64 | 164/428 |  | >0.05 | 1.03 (0.60–1.76) | 0.928 |  |  |
|  |  |  |  |  |  |  |  |  |  |  |  |  |  |  |  |  |  |  |
| Guo | 2015 | China | Chinese | ZNRD1-AS1 | rs3757328 | GG | GA | AA | GG | GA | AA |  | MassARRAY |  |  |  | 7 | 36 |
|  |  |  |  |  |  | 946 | 363 | 35 | 950 | 435 | 51 | 1344/1436 |  | >0.05 | 0.71 (0.46-1.08) | 0.111 |  |  |
|  |  |  |  |  | rs6940552 | GG | GA | AA | GG | GA | AA |  |  |  |  |  |  |  |
|  |  |  |  |  |  | 872 | 413 | 61 | 867 | 495 | 86 | 1346/1448 |  | >0.05 | 0.64 (0.46-0.90) | 0.009 |  |  |
|  |  |  |  |  | rs9261204 | AA | AG | GG | AA | AG | GG |  |  |  |  |  |  |  |
|  |  |  |  |  |  | 821 | 438 | 57 | 819 | 521 | 91 | 1316/1431 |  | >0.05 | 0.64 (0.46-0.90) | 0.009 |  |  |
