# Supplementary material for: Association between genetic variants (rs920778, rs4759314, and rs217727) in LncRNAs and cervical cancer susceptibility in Chinese population: A systematic review and meta-analysis
Source: Front Genet. 2022 Oct 13;13:988207. doi: 10.3389/fgene.2022.988207 (PMC9608570; doi:10.3389/fgene.2022.988207)
Supplement: Supplementary file 2 [file Table2.docx]

**Table 2** Sensitivity analysis for rs4759314 polymorphism and cancer risk..

| **Study** | **OR** | **95%CI** |
| --- | --- | --- |
| Omitting Guo (China 2016) | 0.61 | 0.43-0.85 |
| Omitting Jin (China 2019) | 0.64 | 0.48-0.87 |
| Omitting Weng (China 2018) | 0.57 | 0.42-0.77 |
| Omitting Wu (China 2016) | 0.82 | 0.50-1.35 |
